# New Insights in Genetic Cholestasis: From Molecular Mechanisms to Clinical Implications

**DOI:** 10.1155/2018/2313675

**Published:** 2018-07-26

**Authors:** Eva Sticova, Milan Jirsa, Joanna Pawłowska

**Affiliations:** ^1^Clinical and Transplant Pathology Centre, Institute for Clinical and Experimental Medicine, Prague 4, 140 21, Czech Republic; ^2^Department of Pathology, Third Faculty of Medicine, Charles University, Prague 10, 100 00, Czech Republic; ^3^Laboratory of Experimental Hepatology, Experimental Medicine Centre, Institute for Clinical and Experimental Medicine, Prague 4, 140 21, Czech Republic; ^4^Department of Gastroenterology, Hepatology, Nutritional Disorders and Pediatrics, The Children's Memorial Health Institute (CMHI), Warsaw 04-730, Poland

## Abstract

Cholestasis is characterised by impaired bile secretion and accumulation of bile salts in the organism. Hereditary cholestasis is a heterogeneous group of rare autosomal recessive liver disorders, which are characterised by intrahepatic cholestasis, pruritus, and jaundice and caused by defects in genes related to the secretion and transport of bile salts and lipids. Phenotypic manifestation is highly variable, ranging from progressive familial intrahepatic cholestasis (PFIC)—with onset in early infancy and progression to end-stage liver disease—to a milder intermittent mostly nonprogressive form known as benign recurrent intrahepatic cholestasis (BRIC). Cases have been reported of initially benign episodic cholestasis that subsequently transitions to a persistent progressive form of the disease. Therefore, BRIC and PFIC seem to represent two extremes of a continuous spectrum of phenotypes that comprise one disease. Thus far, five representatives of PFIC (named PFIC1-5) caused by pathogenic mutations present in both alleles of* ATP8B1*,* ABCB11*,* ABCB4*,* TJP2,* and* NR1H4* have been described. In addition to familial intrahepatic cholestasis, partial defects in* ATP8B1*,* ABCB11,* and* ABCB4* predispose patients to drug-induced cholestasis and intrahepatic cholestasis in pregnancy. This review summarises the current knowledge of the clinical manifestations, genetics, and molecular mechanisms of these diseases and briefly outlines the therapeutic options, both conservative and invasive, with an outlook for future personalised therapeutic strategies.

## 1. Introduction

Cholestasis is characterised by an impairment of bile secretion and transport, leading to the subsequent accumulation of toxic bile components in the organism. Bile salts (BS), the main organic solutes in bile, are physiological detergents that facilitate the absorption and transport of lipids, vitamins, and nutrients. BS also play an important role in cell signalling as part of key metabolic processes.

## 2. Bile Salt Synthesis

BS are synthesised from cholesterol in the liver. The size of the total BS pool in human adults is 3-4 g [1, 2]. There are two main pathways of BS synthesis. The classic (neutral) biosynthesis pathway, localised exclusively in the liver and accounting for at least 75% of the total BS pool, starts with modification of the sterol ring, followed by side chain cleavage reactions to synthesise cholic and chenodeoxycholic acid, constituting the primary BS in humans. The first and rate-limiting enzyme in this pathway is microsomal cholesterol 7 alpha-hydroxylase (CYP7A1). In the alternative (acidic) pathway of BS synthesis, side chain oxidation precedes modification of the sterol ring. The first enzyme in the alternative pathway is mitochondrial sterol-27 hydroxylase (encoded by* CYP27A1*). Primary BS are conjugated at the side chain either with taurine or glycine, while water soluble conjugates are excreted into bile where they are rapidly incorporated in mixed micelles containing phospholipids (predominantly phosphatidylcholine) and cholesterol. Thereafter, they are transported into the intestinal tract where they are deconjugated, oxidised, and dehydroxylated to form 7-deoxycholic and lithocholic acid as part of a reaction catalysed by bacterial 7 alpha-dehydroxylases. Finally, most intestinal BS (95%) are reabsorbed in the distal part of the small intestine and transported to the liver via the portal blood and, to a lesser extent, via the hepatic artery in a process known as enterohepatic circulation. The BS pool is recycled 4-12 times a day. BS lost in the faeces (0.2-0.6 g/day) are replenished by* de novo* synthesis in the liver [1–3].

## 3. Bile Salt and Lipid Transporters

**Figure 1:**
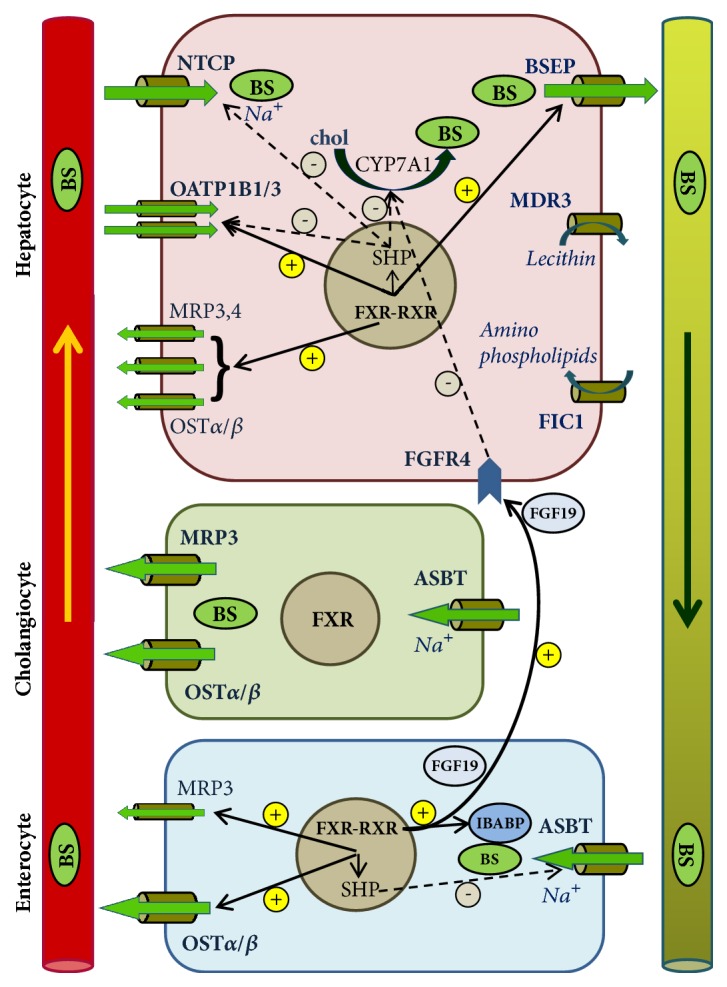
Bile salt and lipid transporters and their regulatory pathways.

Bile acids form dissociated sodium or potassium salts in body fluids with neutral pH. Therefore, BS transporters are designed as anion transporters. Enterohepatic circulation of BS is driven by several specific transport systems expressed predominantly in hepatocytes and biliary and intestinal epithelia (Figure 1).

In hepatocytes, Na+-dependent taurocholate cotransporting peptide (NTCP, also known as the sodium/bile acid cotransporter), encoded by* SLC10A1* (solute carrier family 10 member 1), represents the major conjugated BS uptake system from the blood, contributing 80% of the transport capacity. NTCP is localised in the basolateral (sinusoidal) membrane, and its driving force, the Na^+^ concentration gradient, is maintained by Na^+^/K^+^-ATPase [4, 5]. NTCP deficiency has recently been shown to cause severe familial predominantly conjugated hypercholanemia with no cholestatic jaundice, pruritus, and liver disease [6].

Unconjugated BS are transported by the multispecific ATP- and Na^+^-independent basolateral uptake transporters, organic anion-transporting polypeptide 1B1 (OATP1B1, also termed OATP-C, OATP2, SLC21A6, or LST-1, encoded by* SLCO1B1*), and 1B3 (OATP1B3, synonyms OATP8, SLC21A8, or LST3, encoded by* SLCO1B3*) [4, 7, 8]. They play a secondary role in liver uptake of conjugated BS since the presence of both OATP1Bs only partly compensates for NTCP deficiency [6].

The basolateral domain of hepatocytes also possesses several ATP-dependent efflux pumps, which constitute the multidrug resistance protein (MRP) subfamily (ABCC). ABCC proteins transport organic anions including BS [9, 10].

MRP3 (encoded by* ABCC3*) is a basolateral transporter of BS, glucuronide, and anionic conjugates, including glutathione [11–13]. In the liver, MRP3 is expressed predominantly in the centrilobular hepatocytes, and its expression is low under physiological conditions. MRP3 expression rate is upregulated during cholestasis and independently of any cholestatic manifestation, in individuals with Dubin-Johnson syndrome or after repeated administration of ethinylestradiol [11, 14, 15]. As well as in hepatocytes, Northern blotting of various human tissues indicates the presence of MRP3 in the bile duct epithelium, gallbladder, intestine, pancreas, and kidney [16].

MRP4 (gene* ABCC4*) is an inducible basolateral transporter that cotransports taurine and glycine conjugates of cholic acid with glutathione. It is also a high-affinity transporter of sulphated BS, dehydroepiandrosterone sulphate, eicosanoids, and uric acid, as well as signalling molecules such as cAMP and cGMP [17]. Although MRP4 expression in the liver is low, it can be induced by BS in cholestasis [18, 19]. Strong upregulation of human MRP4 has been demonstrated in patients with BS export pump (BSEP) deficiency [20].

The heterodimeric organic solute transporter OST*α*-OST*β*, initially identified as a basolateral BS efflux system in enterocytes (see below), is also localised in the sinusoidal membrane of hepatocytes [21]. Hepatic OST*α*-OST*β* expression increases in patients with primary biliary cirrhosis and in bile duct-ligated rodents [22]. Thus, during cholestasis, MRP3, MRP4, and OST*α*-OST*β* may provide effective protection against hepatocellular BS overload.

The transfer of BS from the liver to the bile canaliculus is determined by several transport proteins in the canalicular (apical) membrane of hepatocytes, mainly ATP-dependent BSEP and MRPs.

BSEP, encoded by* ABCB11*, is responsible for the ATP-dependent transport of predominantly monovalent conjugated BS across the hepatocyte canalicular membrane. It is exclusively expressed in the liver and localised predominantly in the canalicular microvillar (but not the intermicrovillar) membrane and to a lesser extent in subcanalicular (subapical) vesicles [23]. Expression of BSEP is sensitive to the flux of BS through hepatocytes. The BSEP promoter contains an inverted repeat DNA element (IR-1), a binding site for the farnesoid X receptor (FXR), which plays an important role in maintaining BS homeostasis (see below).

Multidrug resistance protein 3 (MDR3, gene* ABCB4*) is understood to act as a floppase, which translocates phospholipids from the inner to the outer leaflet of the lipid bilayer of the canalicular membrane [24]. Mutations in* ABCB4 *cause cholestasis, which is characterised by the decreased biliary lecithin output, impaired formation of mixed micelles, and the production of more hydrophilic bile with potent detergent properties, resulting in membrane damage [25, 26].

Familial intrahepatic cholestasis type 1 transporter (FIC1, gene* ATP8B1*), a member of the type 4 subfamily of P-type ATPases (P4 ATPase), is a flippase that mediates the translocation of aminophospholipids from the outer (exoplasmic) to the inner (cytoplasmic) leaflet of the plasma membrane [27]. In most eukaryotic cells, phosphatidylcholine and sphingolipids are concentrated in the exoplasmic leaflet, whereas the aminophospholipids (phosphatidylserine and phosphatidylethanolamine) are largely confined to the cytoplasmic leaflet. FIC1 thus helps in maintaining asymmetry and fluidity characteristics of plasma membranes, the essential prerequisites for proper function of transmembrane embedded pumps [27].

Cholangiocytes are important modifiers of bile composition. Conjugated BS need to be actively transported into cholangiocytes via apical sodium-dependent bile acid transporter (ASBT, gene* SLC10A2*) and are exported into the peribiliary capillary plexus via the heterodimeric transporter OST*α*-OST*β* and via MRP3 [28, 29]. Under physiological conditions, BS transporters in cholangiocytes can play a major role in the regulation of intracellular concentrations of BS as signalling molecules. In obstructive cholestasis, cholangiocellular BS receptors may facilitate the removal of BS from stagnant bile [3, 28, 29].

An important step in maintaining BS homeostasis is the reabsorption of BS in the intestinal lumen, predominantly in the ileum. Intestinal epithelial cells reabsorb the majority of secreted BS through ASBT, localised in the brush border membrane of enterocytes. Human ASBT, also called ileal bile acid transporter or ileal sodium-dependent bile acid transporter, consists of 348 amino acids. It transports conjugated and unconjugated BS with a higher affinity for chenodeoxycholic and deoxycholic acid than for taurocholate [30]. After uptake into the enterocyte, the transcellular movement of BS is mediated by cytosolic ileal bile acid-binding protein, also known as fatty acid-binding protein 6 or gastrotropin (IBABP, FABP6, gene* FABP6*), that is cytoplasmically attached to ASBT [30].

The heterodimeric organic solute transporter OST*α*-OST*β* (OST*α*, gene* SLC51A*; OST*β*, gene* SLC51B*) is expressed in the basolateral membrane of enterocytes and effluxes BS to the portal blood. In humans, OST*α*-OST*β* is expressed at high levels in a variety of tissues and is the primary transporter of BS into the systemic circulation [21, 22, 30].

Furthermore, MRP3 may also participate in the basolateral transport of BS in human enterocytes, although its overall contribution is small [10].

## 4. Regulation of Bile Salt Synthesis and Trafficking

The BS biosynthesis and enterohepatic circulation are tightly regulated at many levels but particularly by transcriptional and posttranscriptional mechanisms [2, 3]. A key regulator of BS synthesis and enterohepatic flow is the nuclear farnesoid X receptor (FXR), a major BS-responsive ligand-activated transcription factor with a high affinity for several major endogenous BS [2, 31, 32]. Expression levels of the FXR gene (also known as the* NR1H4* gene—nuclear receptor subfamily 1, group H, member 4) are highest in the intestine, predominantly in the ileal epithelium, liver, and kidneys [31, 32].

FXR acts as an agonist-dependent transcriptional activator of its direct target genes. The preferred DNA-binding sequence for FXR within its target promoters is a variant of inverted repeat-1 motif (IR-1) to which FXR binds as a heterodimer with the retinoid X receptor (RXR). FXR can also downregulate the transcription of specific target genes indirectly via another nuclear receptor, the small heterodimer partner (SHP, the* NR0B2 *gene). SHP is an atypical orphan nuclear receptor without a DNA-binding domain. It acts as a repressor of nuclear receptors, as well as of transcription factors belonging to other protein families [2, 31, 33].

FXR plays a key role in controlling the enterohepatic circulation of BS, largely by directly regulating the expression of several hepatobiliary transporters (Figure 1). In the liver, the heterodimer FXR-RXR induces the expression of BSEP by binding to an IR-1 element at the promoter [34, 35]. Likewise, FXR can directly transactivate the* ABCC2, ABCB4, *and* SLCO1B3 *genes [36, 37].

The FXR-RXR complex directly induces the expression of OST*α*-OST*β* (in ileal enterocytes and in the basolateral membrane of hepatocytes) as well as intestinal expression of the intestinal bile acid-binding protein (IBABP) [22, 37].

In addition to directly activating the main BS efflux systems, under cholestatic conditions, FXR concurrently downregulates the main BS uptake systems, primarily NTCP in the basolateral membrane of hepatocytes and ASBT in the ileal epithelium. OATP1B1 is also suppressed by an FXR-dependent pathway during cholestasis. FXR-mediated repression of target genes is indirect and mainly effected via SHP [2, 33, 37].

As well as regulating transport systems involved in the enterohepatic flow of BS, FXR represses the transcription of genes that encode regulatory enzymes in both classical and alternative BS biosynthetic pathways, predominantly* CYP7A1 *and* CYP27A1 *[2, 33].

There are two important FXR-dependent mechanisms for BS-induced inhibition of* CYP7A1* gene transcription: the FXR/SHP pathway in the liver and the FXR/FGF19/FGFR4 pathway in the intestine [2, 37].

In the liver, the heterodimer FXR/RXR, after binding retinoic acid and BS, induces the expression of SHP. The interaction of SHP with liver receptor homologue-1 (LRH-1, also NR5A2, gene* NR5A2*) and hepatocyte nuclear factor-4*α* (HNF4*α*, also NR2A1, gene* HNF4A*) inhibits* CYP7A1 *transcription [2, 32, 33]. In the* CYP27A1 *gene, the BS response element contains a DNA-binding site for HNF4*α* only, not for LRH-1 [2, 31].

In the intestine, FXR induces an intestinal hormone, fibroblast growth factor 19 (FGF19, gene* FGF19*), which activates hepatic FGF receptor 4 (FGFR4, gene* FGFR4*) and downregulates BS synthesis by inhibiting* CYP71A* expression. The FXR/FGF19/FGFR4 pathway seems to be the physiological mechanism for feedback regulation of BS biosynthesis. Furthermore, the FGF19 autocrine pathway exists in the human liver [2, 31, 37, 38].

Along with FXR-related regulation of BS biosynthesis, there are several FXR-independent mechanisms of* CYP7A1* inhibition mediated by pregnane X receptor (PXR), vitamin D receptor (VDR), the cytokines tumour necrosis factor *α* (TNF*α*) and interleukin 1*β* (IL-1*β*), transforming growth factor *β*1 (TGF*β*1), epidermal growth factor receptor (EGFR), and others. These factors activate signalling pathways, which play roles in protecting against BS toxicity during cholestatic liver injury [2, 31, 32, 37].

Finally, FXR also directly transactivates genes encoding enzymes that metabolise BS, such as human uridine 5'-diphosphate-glucuronosyltransferase 2B4 (UGT2B4, encoded by* UGT2B4*). Interestingly, FXR binds the* UGT2B4* promoter as a monomer without previous heterodimerisation with RXR [32].

Several nuclear receptors such as FXR, RXR, LXR, and SHP are also expressed in cholangiocytes, but their role in bile duct biology remains to be elucidated [29].

## 5. Familial Intrahepatic Cholestasis

Up- and downregulation of transport systems involved in bile formation can explain the impaired liver uptake and excretion of biliary constituents, which result in cholestasis and jaundice in some hereditary and many common acquired liver disorders. Hereditary diseases characterised by hepatocanalicular cholestasis, which is caused by defects in hepatobiliary transporters, their regulator FXR, and in tightness of the liver epithelium, include progressive familial intrahepatic cholestasis (PFIC) types 1 to 5, benign recurrent intrahepatic cholestasis (BRIC) 1 and 2, familial cholelithiasis caused by a lack of biliary secretion of phospholipids (Low Phospholipid-Associated Cholelithiasis or Gallbladder Disease-1), intrahepatic cholestasis of pregnancy (ICP), and several other rare disorders. Hereditary predisposition also plays an important role in drug-induced intrahepatic cholestasis, including cholestasis induced by hormonal contraceptives [39, 40].

### 5.1. Progressive Familial Intrahepatic Cholestasis

PFIC, first described in 1969 [41], is a genetically heterogeneous group of autosomal recessive disorders caused by mutations in genes that encode hepatocanalicular transporters of BS and phospholipids, their regulator FXR, and TJP2 which is essential for tightness of cell junctions between the epithelial cells lining the bile ducts. The exact incidence of PFIC is not known, but it is estimated to be in the region of 1/50000 to 1/100000 births. Both genders are equally affected [42]. Clinically, PFIC usually manifests in the first year of life and is characterised by jaundice, severe pruritus, hepatosplenomegaly, steatorrhoea, and retardation of growth and mental development. Further symptoms caused by a deficit of fat-soluble vitamins include coagulopathy, osteopaenia, and neuromuscular disorders. Without adequate treatment, the disease progresses to liver fibrosis and cirrhosis and usually ends in death due to liver failure in the first or, more rarely, in the second decade of life [40, 42].

The PFIC group consists of five representatives (PFIC 1-5), which are classified into two main categories according to levels of serum *ɣ*-glutamyl transferase (GGT) activity. GGT is considered a cholestatic enzyme and, when elevated, is associated with damage to the apical membranes of bile ducts and the disruption of intercellular connections due to high concentrations of BS in bile. However, in some cholestatic diseases, synthesis or canalicular secretion of BS is virtually absent and there are no conditions that predispose either to the release of membrane GGT from damaged cholangiocytes or to leakage of bile into the extracellular space and subsequently the blood. This explains why, in hereditary cholestasis, GGT activity is increased only in disorders of phospholipid secretion (PFIC3).

#### 5.1.1. Progressive Familial Intrahepatic Cholestasis Type 1 (PFIC1)

PFIC1 (*ATP8B1* disease, OMIM #211600), formerly Byler's disease [41], is the consequence of a severe defect in the gene encoding ATPase,* ATP8B1/FIC1* (familial intrahepatic cholestasis 1), localised in the long arm of the 18th chromosome (18q21) [43, 44]. ATP8B1 deficiency, which causes membrane phospholipid asymmetry of the canalicular membrane, reduces the capacity of the liver to secrete bile [45, 46]. In addition to the previously described common symptoms of PFIC, clinical manifestation of PFIC1 also includes a wide range of extrahepatic symptoms. Extrahepatic manifestations of* ATP8B1* disease include elevated sweat chloride concentrations, delayed pubescence and growth, and watery diarrhoea as well as impaired hearing and/or pancreatitis [47]. These symptoms often persist, while diarrhoea even tends to worsen after liver transplantation (LTX). Liver allografts in PFIC1 patients may display diffuse steatosis with a variable necroinflammatory component, with subsequent fibrosis [48]. Both diarrhoea and steatosis/steatohepatitis may improve after biliary diversion [49].

Laboratory findings with regard to PFIC1 have reported cholestasis with low serum levels of GGT, increased serum concentrations of primary BS, and normal levels of cholesterol. Aminotransferases are initially within the reference range, but during disease progression they gradually increase by up to tenfold [41]. Histopathological changes predominantly involve canalicular cholestasis accentuated around the central veins. The interlobular ducts can be hypoplastic due to subnormal bile flow. Giant-cell changes of hepatocytes are generally not observed in biopsies. Liver fibrosis progression corresponds with the respective stage of the disease and can terminate in cirrhosis. A typical ultrastructural finding is the presence of Byler-type coarsely granular bile in the bile canaliculi [50]. Molecular diagnosis is based on the detection of pathogenic mutations in both alleles of the* ATP8B1* gene. The milder intermittent nonprogressive form of* ATP8B1* deficiency is called BRIC1 (see below).

#### 5.1.2. Progressive Familial Intrahepatic Cholestasis Type 2 (PFIC2)

PFIC2 (*ABCB11* disease, OMIM #601847) is caused by mutations in* ABCB11*, which is located on the long arm of the second chromosome (2q24) and encodes the canalicular transport protein ABCB11/BSEP [51]. Clinical and laboratory findings with regard to PFIC2 are similar to PFIC1 but extrahepatic symptoms are not present. Additionally, in PFIC2 the formation of gallstones and the early elevation of serum aminotransferase activity can occur. The development of hepatobiliary malignancies, both hepatocellular carcinoma and cholangiocarcinoma, can be a serious complication of PFIC2, which is not observed in PFIC1 [52, 53]. Screening for liver tumours is recommended from the first year of life in PFIC2 patients.

Except for PFIC2, mutations in* ABCB11* may result in a milder nonprogressive form of PFIC2 known as BRIC2 as well as other forms of cholestasis such as ICP and/or drug-induced cholestasis (see below).

**Figure 2:**
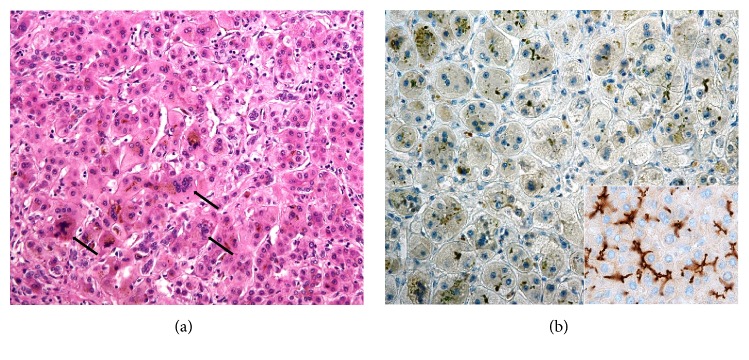
*Histopathology of ABCB11 disease*. (a) Giant-cell hepatitis (arrows) with hepatocanalicular cholestasis and (b) complete absence of ABCB11/BSEP protein are typical findings in PFIC2 patients. Inset: immunohistochemical positivity of BSEP in the apical (canalicular) domain of hepatocytes in a healthy control. (a) Hematoxylin and eosin, original magnification x200. (b, inset) Immunohistochemical staining with ABCB11 Rabbit Polyclonal Antibody (NBP1-89319, Novus Biologicals, USA), original magnification x200 (b), x400 (inset).

Histologically, the typical findings in the liver biopsies of PFIC2 patients are giant-cell (syncycial) hepatitis with hepatocanalicular cholestasis, while extramedullary haemopoiesis is frequently discernible within lobules. Interlobular bile ducts can be hypoplastic, and ductular proliferation at the peripheries of portal triads is commonly observed. The ABCB11/BSEP protein is usually undetectable immunohistochemically (Figure 2). Ultrastructurally, canalicular bile can be either amorphous or filamentous but is not coarsely granular [51].

Untreated PFIC2 usually leads to progressive liver fibrosis and cirrhosis, the development of which is more rapid than in PFIC1. One curative method for patients with PFIC2 is LTX. Unlike in PFIC1, steatosis and steatohepatitis are not present in liver allografts in the case of PFIC2. However, in some patients, a recurrence of the PFIC2 phenotype, accompanied by elevated serum levels of BS and bilirubin with almost normal GGT activity, has been observed after LTX [54, 55]. It has been shown that canalicular immunostaining of the BSEP, MDR3, and MRP2 proteins is preserved in the liver graft biopsies and no discernible changes in BSEP immunoreactivity distribution between the apical membrane and the cytoplasm have been reported; nonetheless,* de novo* polyclonal inhibitory antibodies directed against the first extracellular loop of BSEP have been observed in the posttransplant serum of patients, a condition known as Autoimmune BSEP Disease (AIBD) [56, 57]. The exact mechanism by which BS transport is inhibited by anti-BSEP antibodies is still unknown. It is supposed that the cross-linking of BSEP molecules, which in turn disrupts the structure and function of canalicular membranes, and/or the direct mechanical occlusion of the BSEP pore, might explain the inhibitory effects of the antibodies [56, 57]. It is estimated that up to 8% of transplanted PFIC2 patients develop anti-BSEP antibodies [56]. This may be explained by the insufficient autotolerance against BSEP exhibited in some patients with severe* ABCB11* mutations, resulting in the complete absence of the BSEP protein. The causal relationship between recurrent cholestasis in the liver grafts of PFIC2 patients and the occurrence of* de novo* antibodies directed against BSEP is further supported by observations that plasmapheresis and the administration of anti-CD20 antibodies (rituximab) may alleviate symptoms of cholestasis [54–57].

Interestingly, posttransplant development of inhibitory autoantibodies directed against FIC1 and MDR3 transporters with corresponding phenotypes has not yet been documented in patients with PFIC1 and PFIC3, respectively [44].

#### 5.1.3. Progressive Familial Intrahepatic Cholestasis Type 3 (PFIC3)

PFIC3 (*ABCB4 *disease, OMIM #602347) is caused by mutations in both alleles of the* ABCB4/MDR3* gene that encode phospholipid transporter MDR3, expressed in the canalicular (apical) membrane of hepatocytes [26]. As well as PFIC3, there is evidence that either biallelic or monoallelic* ABCB4* defects may cause or predispose patients to a wide spectrum of human liver diseases, such as Low Phospholipid-Associated Cholelithiasis Syndrome (LPAC, OMIM #600803), Intrahepatic Cholestasis of Pregnancy (ICP, OMIM #147480), drug-induced liver injury, transient neonatal cholestasis, small duct sclerosing cholangitis, and adult biliary fibrosis or cirrhosis [58–61]. Moreover, hepatocellular carcinoma and intrahepatic cholangiocarcinoma have been documented in patients with* ABCB4/MDR3 *mutations [61].

Clinical and laboratory findings associated with PFIC3 correspond to the other two forms of PFIC, but are characterised by the absence of extrahepatic symptoms (except for cholelithiasis). Unlike PFIC1 and PFIC2, elevated serum GGT activity and normal cholesterol levels are typically observed, as exhibited by the MDR3 deficit [26, 62].

Histopathological findings in connection with* ABCB4* disease are variable. Syncytial (giant-cell) changes, cholestasis, portal inflammatory infiltrate, and periportal ductular proliferation accompanied by various stages of fibrosis have been observed in liver tissue. Lipid crystals within bile ducts and fibroobliterative bile duct lesions can be seen. Immunohistochemistry has indicated the absence of the canalicular MDR3 protein, predominantly in early onset forms; however, its use is limited compared to PFIC2. Ultrastructurally, bile is dense and amorphous in PFIC3 [26, 61].

Prolonged cholestasis in PFIC3 is associated with significant accumulation of copper in liver tissue and with increased urine copper excretion, i.e., findings that overlap with the diagnostic criteria for Wilson's disease [63, 64].

#### 5.1.4. Progressive Familial Intrahepatic Cholestasis Type 4 (PFIC4)

The fourth form of PFIC (PFIC4,* TJP2* deficiency, OMIM #615878), first described in 2014 [65], is clinically similar to PFIC2 and caused by homozygous or compound heterozygous mutations in the* TJP2* gene, located in the 9q12 chromosome. Tight junction protein 2 (TJP2, also zona occludens-2) is a cytoplasmic component of cell-cell junctional complexes expressed in most epithelia and creates a link between transmembrane tight junction proteins and the actin cytoskeleton. Complete TJP2 deficiency is associated with a significant reduction in an integral tight junction protein, claudin-1, predominantly in the canalicular membranes of liver cells, which subsequently leads to the disruption of intercellular connections and the leakage of bile through the paracellular space into the liver parenchyma [65, 66].

Patients with* TJP2* deficiency display severe progressive cholestatic liver disease in early childhood, which puts them at increased risk of developing hepatocellular carcinoma [67]. Serum GGT activity is normal or, at most, slightly increased, while expression of the canalicular proteins BSEP and MDR3 is maintained. In addition to liver impairment, extrahepatic features have been identified in PFIC4 patients, including neurological and respiratory disorders [66]. A single homozygous missense mutation in* TJP2* has been previously described as causing benign familial hypercholanemia, a rare disorder of oligogenic inheritance, which usually manifests in elevated serum BS concentrations, pruritus, and fat malabsorption, but which does not lead to the development of liver disease [68].

#### 5.1.5. Progressive Familial Intrahepatic Cholestasis Type 5 (PFIC5)

PFIC5 (OMIM #617049), first described in 2016 [69], is a cholestatic disorder caused by mutations in* NR1H4*, a gene located at 12q23.1, which encodes FXR, the key regulator of BS metabolism (see above). Homozygous loss of FXR function is associated with severe neonatal cholestasis and early onset vitamin K-independent coagulopathy, which rapidly progresses to end-stage liver disease. The nonresponsiveness of coagulopathy to vitamin K treatment is likely a direct consequence of the loss of FXR function, representing an important distinguishing diagnostic feature of* NR1H4*-related cholestasis. In addition to low (or normal) GGT activity, serum levels of alpha-fetoprotein are typically elevated [69].

Liver biopsies have shown diffuse giant-cell transformation of hepatocytes with hepatocellular cholestasis and ductular proliferation. Progressive fibrosis and even micronodular cirrhosis are evident at later stages. BSEP is undetectable in the apical domains of hepatocytes, which is consistent with low activity of GGT; MDR3 expression, on the other hand, is maintained [69].

Since FXR affects several metabolic pathways, some* NR1H4* variations may be associated with susceptibility to various human diseases.* NR1H4* single-nucleotide polymorphisms seem to be correlated with differences in glucose homeostasis, gallstone formation, ICP, inflammatory bowel disease, and several other disorders [70, 71].

### 5.2. Benign Recurrent Intrahepatic Cholestasis (BRIC)

BRIC is a group of genetically heterogeneous autosomal recessive diseases, characterised by intermittent episodes of cholestasis. They are caused by mutations in the* ATP8B1* and* ABCB11* genes (the same as in PFIC1 and PFIC2) and probably in at least one other as yet unidentified gene [72]. There are two genetically characterised forms of BRIC: BRIC1 (Summerskill-Walshe-Tygstrup syndrome, OMIM #243300) and BRIC2 (OMIM #605479), caused by partial deficiency in ATP8B1 [39, 73] and ABCB11 [74], respectively. The disease typically manifests before the second decade of life, but the age of the first manifestation can vary greatly. The same variability has been documented in the duration of cholestatic episodes (several days to several months) and their intensity. Infection or pregnancy may act as a triggering factor. The clinical manifestations include jaundice, pruritus, fatigue, anorexia, and steatorrhoea. Increase of bilirubin and BS levels in serum has been observed during cholestatic attacks, but GGT activity and serum cholesterol levels tend to be normal. Aminotransferase activity is usually normal or only slightly elevated, while biochemical parameters fall within normal ranges. Liver biopsies performed during cholestatic attacks have demonstrated hepatocanalicular cholestasis without fibrosis. During the asymptomatic period, the histological picture is completely normal. Since canalicular expression of BSEP protein in hepatocytes is mostly maintained, immunohistology is of limited significance in these cases. Diagnosis of BRIC1 and BRIC2 is based on demonstrating evidence of mutations in both alleles of the corresponding genes,* ATP8B1* and* ABCB11*.

While mutations causing PFIC are often located in the conserved regions of the genes that encode conserved functional domains of the corresponding proteins, mutations in BRIC only partially impact protein function and expression. However, several cases of initially benign episodic cholestasis that have subsequently transitioned to a persistent progressive form of the disease have been reported [75]. Thus, BRIC and PFIC seem to represent two extremes of a continuous spectrum of phenotypes of the one disease.

The administration of statins, corticosteroids, cholestyramine, or ursodeoxycholic acid (UDCA) is usually not very effective in BRIC patients. Improvement of pruritus and shortening of symptomatic phase has been described in BRIC patients treated with the antibiotic rifampicin, a potent human activator of PXR [76, 77]. However, severe hepatotoxicity after long-term administration of rifampicin has been reported in patients with cholestatic disorders [77, 78]. Nasobiliary drainage seems to have a prompt effect during cholestatic episodes, but the mechanism of action remains unclear [79].

### 5.3. Intrahepatic Cholestasis of Pregnancy (ICP)

ICP (OMIM #147480), also known as obstetric cholestasis, is characterised by cholestasis and pruritus with onset in pregnancy, usually in the third trimester. It is associated with abnormal liver function in the absence of other liver diseases and resolves completely after delivery [80, 81]. The incidence of ICP varies with geographical location and ethnicity. Seasonal variations indicate higher incidence in winter months, as reported in some countries, and it more commonly occurs in association with multiple pregnancies and following in vitro fertilisation. In Europe, ICP affects about 1% of all pregnancies [81]. A typical symptom is intense pruritus, commonly localised in the palms and the soles, which progresses during pregnancy. Elevated levels of serum BS and increased liver aminotransferase activity are typical laboratory findings in such cases. Increased serum bilirubin levels have been observed in a small proportion of cases, while jaundice only rarely occurs in affected women [81].

ICP usually resolves soon after delivery. Although the disease is considered relatively benign for the mother, there is an increased rate of adverse foetal outcomes, including foetal distress, foetal asphyxia, stillbirth, or even intrauterine death: all known complications of ICP [81, 82]. ICP may also be associated with an abnormal metabolic profile in afflicted women, especially higher prevalence of dyslipidaemia, impaired glucose tolerance, and maternal comorbidities, e.g., gestational diabetes and preeclampsia [82, 83]. Administration of UDCA improves clinical symptoms, predominantly pruritus, and may even reduce the risk of premature birth [84–86].

Mutations in* ABCB4, ABCB11, *and* ATP8B1* have been identified in some patients suffering from ICP [87–90]. Additionally, variations in* NR1H4* may be implicated in ICP, possibly via downregulation of BSEP expression [71].

## 6. Conclusions and Perspectives

Identification of the genes involved in hereditary cholestasis has advanced our understanding of the molecular mechanisms behind bile formation and transport.

Recently, mutations in* TJP2* and* NR1H4* have been found in patients with apparent BSEP deficiency, normal serum GGT activity, and no mutations in* ABCB11*. However, there are rare cases of genetic cholestasis causes of which have yet to be identified. Therefore it seems likely that other yet unidentified genes involved in secretion and/or transport of cholephiles may be responsible for FIC-like phenotypes.

Current therapeutic regimens in FIC patients comprise both nonsurgical and surgical approaches. Nonsurgical therapy includes drugs, predominantly UDCA and rifampicin, and nasobiliary drainage, the latter being predominantly used in BRIC patients with intractable pruritus during long-lasting cholestatic episodes [76, 77, 79]. However, the mechanism responsible for the instant and complete relief from pruritus is still unclear.

In addition to cholestyramine, phenobarbital, S-adenosylmethionine, opiate antagonists, UDCA, rifampicin, and serotonin antagonists and its reuptake inhibitors seem to be beneficial in managing refractory pruritus in patients with PFIC [77, 91, 92]. Furthermore, clinical and biochemical improvements have been observed in BRIC patients with intractable pruritus after using the molecular adsorbent recirculating system (MARS) and the Prometheus system, an extracorporeal liver support therapy based on fractionated plasma separation and adsorption [93].

Surgical therapies include partial biliary diversion, i.e., mechanical interruption of BS enterohepatic circulation in order to prevent reabsorption of pruritogens (unidentified thus far) and their precursors, which is primarily useful in noncirrhotic children with low-GGT PFIC, permanent cholestasis and/or intense refractory pruritus, and orthotopic LTX, which is still the ultimate treatment modality for many PFIC patients [91]. However, as well as the risks associated with these invasive procedures, other serious postoperative-related complications have been reported, particularly in PFIC1 patients with extradigestive symptoms, which can persist or even worsen, most likely due to extrahepatic expression of FIC1 [47]. Moreover, the development of steatosis and steatohepatitis leading to progressive liver allograft fibrosis in PFIC1 patients [48] and recurrence of the PFIC2 phenotype in* ABCB11* deficiency may represent other serious complications after LTX, and for some patients may even necessitate retransplantation [54–57].

Importantly, because of the shortage of cadaveric donors, parental living-related LTX is often considered in PFIC patients. As heterozygous conditions can cause mild-to-moderate disease expression under certain circumstances, the use of blood relatives as donors is seen as controversial. In such cases, it is likely that the heterozygous status of the donor allograft could pose a risk of complications during the late posttransplant period, such as lithiasis or cholestasis, which can be precipitated by infections or by drugs administered to the patient. However, the results of a recent study indicate that the PFIC heterozygote status of the organ donor does not increase the risk of liver dysfunction [94].

The limited effectiveness of current conservative procedures along with the complications of invasive methods, particularly LTX, stress the need for the development and introduction of new treatment options. Total biliary diversion, pharmacological diversion of BS, and hepatocyte transplantation as well as gene- and mutation-targeted pharmacotherapies represent promising future therapeutic approaches. The latter two methods provide the basis for personalised treatment strategies in FIC patients. Pharmacological chaperones (e.g., 4-phenylbutyrate), agonists of nuclear receptors (e.g., statins, fibrates, 6-ethyl chenodeoxycholic acid), endoplasmic reticulum-associated degradation inhibitors (MG132), and several other mutation-specific drugs can be used to increase the expression of functional proteins and mitigate or even eliminate deficient phenotypes [95–97].

Nevertheless, despite the indisputable progress in the therapy of FIC patients, the increased risks of hepatobiliary malignancies, particularly in relation to* ABCB11* deficiency, still remain serious life-threatening complications. Further studies are required in order to elucidate the complex pathogenesis of cholestasis, unlock the mechanism of cholestatic pruritus, and improve the clinical management of both hereditary and acquired cholestatic liver diseases.

## Abbreviations

ABC: ATP-binding cassette subfamily
ABCB4: ATP-binding cassette, subfamily B, member 4
ABCB11: ATP-binding cassette, subfamily B, member 11
AIBD: Autoimmune BSEP Disease
ASBT: Apical sodium-dependent bile acid transporter
ATP8B1: ATPase, aminophospholipid transporter, class I, type 8B, member 1
BRIC: Benign Recurrent Intrahepatic Cholestasis
BS: Bile salts
BSEP: Bile salt export pump
CYP7A1: Cholesterol 7 alpha-hydroxylase
CYP27A1: Sterol-27 hydroxylase
FGF19: Fibroblast growth factor 19
FGFR4: Fibroblast growth factor receptor 4
FIC: Familial intrahepatic cholestasis
FXR: Farnesoid X-activated receptor
GGT: *ɣ*-Glutamyl transferase
HNF4*α*: Hepatocyte nuclear factor 4*α*
IBABP: Intestinal bile acid-binding protein
ICP: Intrahepatic cholestasis of pregnancy
IR-1: Inverted repeat DNA element with 1 nucleotide spacing
LPAC: Low Phospholipid-Associated Cholelithiasis Syndrome
LTX: Liver allograft transplantation
LXR: Liver X receptor
MDR3: Multidrug resistance protein 3
MRP: Multidrug resistance-associated protein
NR1H4: Nuclear receptor subfamily 1, group H, member 4
NTCP: Na+-taurocholate cotransporting polypeptide/solute carrier family 10 member 1
OATP: Organic anion-transporting polypeptide
OMIM: Electronic database Online Mendelian Inheritance in ManTM
OST: Organic solute transporter
PFIC: Progressive Familial Intrahepatic Cholestasis
PXR: Pregnane X receptor
RXR: Retinoid X receptor
SHP: Small heterodimer partner
SLC22A: Solute carrier family 22 (organic cation transporter), member 1
SLCO: Solute carrier organic anion transporter family
TJP2: Tight junction protein 2; zona occludens-2
UDCA: Ursodeoxycholic acid
UGT2B4: Uridine 5′-diphosphate-glucuronosyltransferase 2B4.
